# Antitumor Activity of *Fucus vesiculosus*-Derived Phlorotannins through Activation of Apoptotic Signals in Gastric and Colorectal Tumor Cell Lines

**DOI:** 10.3390/ijms22147604

**Published:** 2021-07-16

**Authors:** Marcelo D. Catarino, Iva Fernandes, Hélder Oliveira, Mylene Carrascal, Rita Ferreira, Artur M. S. Silva, Maria Teresa Cruz, Nuno Mateus, Susana M. Cardoso

**Affiliations:** 1 LAQV-REQUIMTE, Department of Chemistry, University of Aveiro, 3810-193 Aveiro, Portugal; mcatarino@ua.pt (M.D.C.); ritaferreira@ua.pt (R.F.); artur.silva@ua.pt (A.M.S.S.); 2 REQUIMTE/LAQV, Department of Chemistry and Biochemistry, Faculty of Sciences, University of Porto, 4169-007 Porto, Portugal; iva.fernandes@fc.up.pt (I.F.); hjcoliv@gmail.com (H.O.); nbmateus@fc.up.pt (N.M.); 3 CNC.IBILI, Faculty of Pharmacy, Health Sciences Campus, University of Coimbra, 3000-548 Coimbra, Portugal; mylenecarrascal87@gmail.com (M.C.); trosete@ff.uc.pt (M.T.C.)

**Keywords:** *Fucus vesiculosus*, phlorotannins, antitumor, gastric cancer, colorectal cancer, cell cycle arrest, apoptosis, UHPLC, flow cytometry

## Abstract

Seaweeds are one of the largest producers of biomass in the marine environment and a source of multiple bioactive metabolites with valuable health benefits. Among these, phlorotannins have been widely recognized for their promising bioactive properties. The potential antitumor capacity of *Fucus vesiculosus*-derived phlorotannins remains, however, poorly explored, especially in gastrointestinal tract-related tumors. Therefore, this work aimed to evaluate the cytotoxic properties and possible mechanisms by which *F. vesiculosus* crude extract (CRD), phlorotannin-rich extract (EtOAc), and further phlorotannin-purified fractions (F1–F9) trigger cell death on different tumor cell lines of the gastrointestinal tract, using flow cytometry. The results indicate that *F. vesiculosus* samples exert specific cytotoxicity against tumor cell lines without affecting the viability of normal cells. Moreover, it was found that, among the nine different phlorotannin fractions tested, F5 was the most active against both Caco-2 colorectal and MKN-28 gastric cancer cells, inducing death via activation of both apoptosis and necrosis. The UHPLC-MS analysis of this fraction revealed, among others, the presence of a compound tentatively identified as eckstolonol and another as fucofurodiphlorethol, which could be mainly responsible for the promising cytotoxic effects observed in this sample. Overall, the results herein reported contribute to a better understanding of the mechanisms behind the antitumor properties of *F. vesiculosus* phlorotannin-rich extracts.

## 1. Introduction

Cancer is the second global leading cause of death and, according to the World Health Organization (WHO), in 2020, it was responsible for nearly 10 million deaths. Among the most common cancers, colorectal and stomach cancers occupy the third and fifth places of the leading types of cancer in 2020, respectively. However, when it comes to the most frequent causes of cancer death, they appeared in the second and fourth places in the same year, respectively [1]. Poor behavioral and dietary habits including tobacco use, alcohol use, lack of fruit and vegetable intake, and physical inactivity are the most common risk factors that will lead to the development of these two types of cancer. Moreover, infection by *Helicobacter pylori* is one of the most important risk factors for the development of stomach cancer, while inflammatory bowel disease, including Chron’s disease and ulcerative colitis, is also a major factor in the development of colorectal cancer [2,3].

In this field, phlorotannins, i.e., phloroglucinol-based phenolic compounds exclusively from brown algae, have drawn much attention during recent years thanks to their promising bioactive properties, particularly their antitumor potential evidenced against different types of cancer [4]. Induction of apoptosis is one of the most common mechanisms activated by these compounds, being described for phlorotannins extracted from multiple seaweed species and in numerous tumor cell lines including HepG2 and Hep3B human hepatoma, MCF-7 human breast cancer, S180 murine sarcoma, SKOV3 human ovarian cancer, and PancTu1 human pancreatic cancer, among several others [5,6,7,8,9,10]. As it happens, compounds such as phloroglucinol, eckol, dieckol, and eckstolonol have been repeatedly reported for their abilities to stimulate the expression of distinct pro-apoptotic proteins, including several caspases (-3, -7, -8, and -9), cytochrome *c,* Bid and Bim, Bak and Bax, and p53 [5,6,9,11,12,13,14], while inhibiting the expression of anti-apoptotic proteins such as X-linked inhibitor of apoptosis protein (XIAP), FLICE-like inhibitory protein (FLIP), Bcl-2, and Bcl-x_L_ [6,9,12,14].

However, considering that the main entrance route of phlorotannins in the human organism is via oral ingestion, for these compounds to exert such in vivo effects on the cancer types mentioned before, they must be able to resist the digestive process, cross the intestinal barrier, enter the blood stream, and be delivered to the target tissues. Recent studies have shown that, like plant phenolics, phlorotannins tend to degrade during their passage throughout the gastrointestinal tract, and only a small portion will reach the intestinal lumen intact and become bioaccessible for further absorption [15]. Nevertheless, because the gastrointestinal tract will be in direct contact with phlorotannins, this would be the most probable target for benefiting from the potential bioactivities of these phenolics, including their antitumor effects, and thus a possible therapeutic strategy for preventing the development of the multiple tumors that may occur along the digestive tract. In this regard, several authors have reported promising results related to the anti-proliferative effects of phlorotannin extracts from different seaweeds against distinct colon cell lines. Indeed, Nwosu et al. [16] observed a dose-dependent anti-proliferative effect on Caco-2 cells treated either with *Ascophyllum nodosum* or *Alaria esculenta* phlorotannin-rich extracts. Likewise, the 60% ethanol extract of *F. evanescens* containing 10.1% dry weight (dw) of phenolic compounds was found to inhibit the proliferation of DLD-1 and HT-29 human colorectal adenocarcinoma cells in 67 and 63%, respectively [17]. More recently, Montero et al. [18] found that ethanol extracts from *S. muticum* collected along the North Atlantic coast during the same harvesting season exhibited different anti-proliferative activities towards HT-29 cells according to their total phlorotannin content, i.e., the extracts from samples collected in Norway, which had the highest phlorotannin contents (approximately 5 mg/g extract), displayed growth inhibitions almost twice as strong as those observed for samples from Portugal, which contained approximately 4 mg/g extract, thus supporting the causality link between the extracts’ phlorotannins content and their anti-proliferative effects.

The existing literature on the potential antitumor capacity of *F. vesiculosus*-derived phlorotannins remains, however, quite underexplored, especially in tumor cell lines of the gastrointestinal tract. Moreover, little is known about the mechanisms behind the anti-proliferative activity produced by the phlorotannins from this species. Therefore, this work aimed to explore the cytotoxic properties as well as the possible mechanisms by which *F. vesiculosus* crude extract (CRD), phlorotannin-rich extract (EtOAc), and further phlorotannin-purified fractions may activate cell death on different tumor cell lines of the gastrointestinal tract. In addition, characterization of the sample showing the strongest antitumor activity was carried out as an attempt to unveil the possible compounds responsible for such effects.

## 2. Results and Discussion

### 2.1. Effect of CRD, EtOAc, and Purified Fractions on Tumor Cells’ Viability

**Figure 1:**
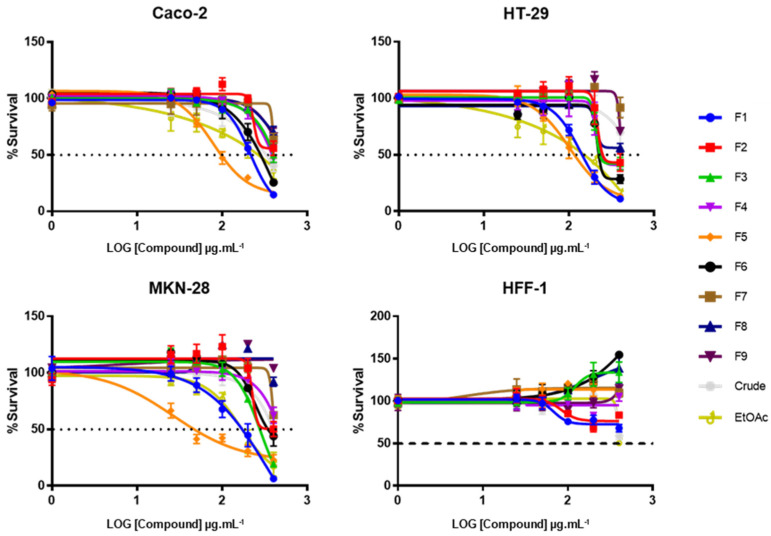
Effect of *F. vesiculosus* samples on the cell viability of Caco-2, HT-29, MKN-28, and HFF-1 cells after 48 h. Data are expressed as percentage of survival compared with the negative control and are presented as the mean ± SEM of at least three independent experiments.

To determine the effects of *F. vesiculosus* CRD, EtOAc, and subsequent subfractions on the viability of gastric and colon tumor cells, serial dilutions of each were prepared and added to the culture medium of MKN-28, Caco-2, and HT-29 cells. To verify the specificity of the cytotoxic effect of the samples towards cancer cells, the same procedure was also carried out on a normal cell line of human fibroblast (HFF-1). Overall, CRD, EtOAc, and following subfractions presented a dose-dependent cytotoxic effect against the three tumor cell lines tested (Figure 1). Among them, F7, F8, and F9 were the least active, as none of these three was able to reduce the cell viability below 50%. On the contrary, F5 displayed the best overall cytotoxic effect against all three tumor cell lines (IC_50_ values of 56.3 ± 14.7 µg/mL, 97.4 ± 11.6 µg/mL, and 118.8 ± 19.7 µg/mL for MKN-28, Caco-2, and HT-29, respectively), followed by EtOAc and F1, which also presented low IC_50_ values (161.7 ± 16.1 and 173.0 ± 33.8 µg/mL for MKN-28, 257.2 ± 32.3 and 219.5 ± 17.1 µg/mL for Caco-2, and 170.0 ± 2.8 and 146.8 ± 13.1 µg/mL for HT-29, respectively). When evaluated for their cytotoxic effect on HFF-1 cells, none of the samples affected cell survivability below 50% for any of the concentrations tested. Interestingly, according to the curves observed in Figure 1, some samples (F3, F6, and F8) even appear to produce a proliferative effect on this cell line, although these observations are most likely resultant from a stimulatory effect of the mitochondrial activity rather than in the growth rate. Regardless, the overall results suggest that these *F. vesiculosus* samples, particularly EtOAc, F1, and F5, display specific cytotoxic effects against tumor cells without critically affecting the viability of normal cells.

The antiproliferative properties of phlorotannins and phlorotannin-rich extracts is a subject that has already been addressed by several authors. Indeed, phlorotannin extracts from *Alaria esculenta*, *Ascophyllum nodosum*, *Laminaria japonica*, *Sargassum muticum*, and *Bifurcaria bifurcate*, among others, were shown to dose-dependently reduce the cell proliferation of numerous tumor cell lines such as Caco-2, HT-29, human hepatoma (BEL-7402), mouse leukemia (P388), and mouse teratocarcinoma (ATDC5) [16,18,19,20,21]. Studies focusing the antitumor activity of phlorotannins from the genus *Fucus* are, however, quite scarce, although some authors have already evidenced promising results. According to Geisen et al. [10], a strong dose-dependent cytotoxic effect of a hydrophilic fraction isolated from a *F. vesiculosus* 70% acetone extract was achieved on four different pancreatic cancer cell lines (Panc1, Panc89, PancTU1, and Colo357), with IC_50_ ranging from 17.35 to 28.9 µg/mL after 72 h of exposure. Additional research from this group revealed that, after further fractionation of a *F. vesiculosus* acetone extract, the two most active fractions against Panc89 and PancTU1 cells presented a characteristic ^1^H NMR fingerprint of two molecules belonging to the polyphenols group, although they could not achieve their full chemical structure [22]. In turn, a phlorotannin-rich EtOAc fraction from a dichloromethane/methanol (1:1) extract of *F. spiralis* was reported to induce a significant decrease on the survival rate of human cervical adenocarcinoma (HeLa), colorectal adenocarcinoma (LS-174T), and lung carcinoma (A549) cells, without affecting the survivability of the normal human lung fibroblasts (MRC-5) [23]. Likewise, a study carried out with a crude polyphenolic fraction of *E. cava* revealed that the cytotoxic effects observed on two human leukemia cell lines (THP-1 and U-937), a murine colon cancer cell line (CT-26), and a mouse melanoma cell line (B-16) were not present on the normal Chinese hamster fibroblast cell line (V79-4) [24]. These studies are, therefore, in agreement with the results herein attained, thus supporting the hypothesis that the *F. vesiculosus* samples obtained in this work, particularly EtOAc, F1, and F5, may exert a specific cytotoxic effect against tumor cells, without affecting normal cells.

### 2.2. Effects on Cell Cycle after Exposure to F. vesiculosus Samples

**Figure 2:**
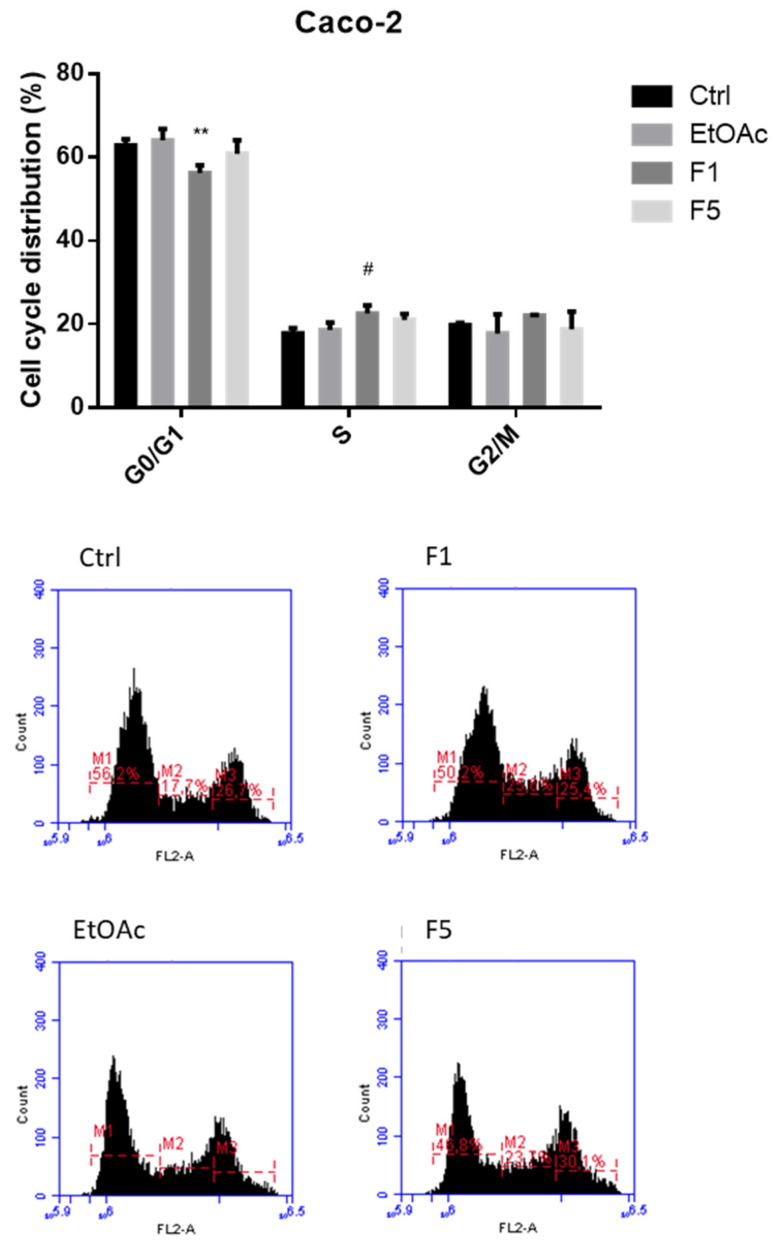
Effect of *F. vesiculosus* samples on the cell cycle distribution of Caco-2 cells after 48 h. Data are expressed as percentage of propidium iodide positive (PI^+^) cells and are presented as the mean ± SEM of at least three independent experiments (* *p* < 0.05, ** *p* < 0.01, when compared with control; the symbols * and # were used to express statistical significance within G0/G1 and S, respectively).

**Figure 3:**
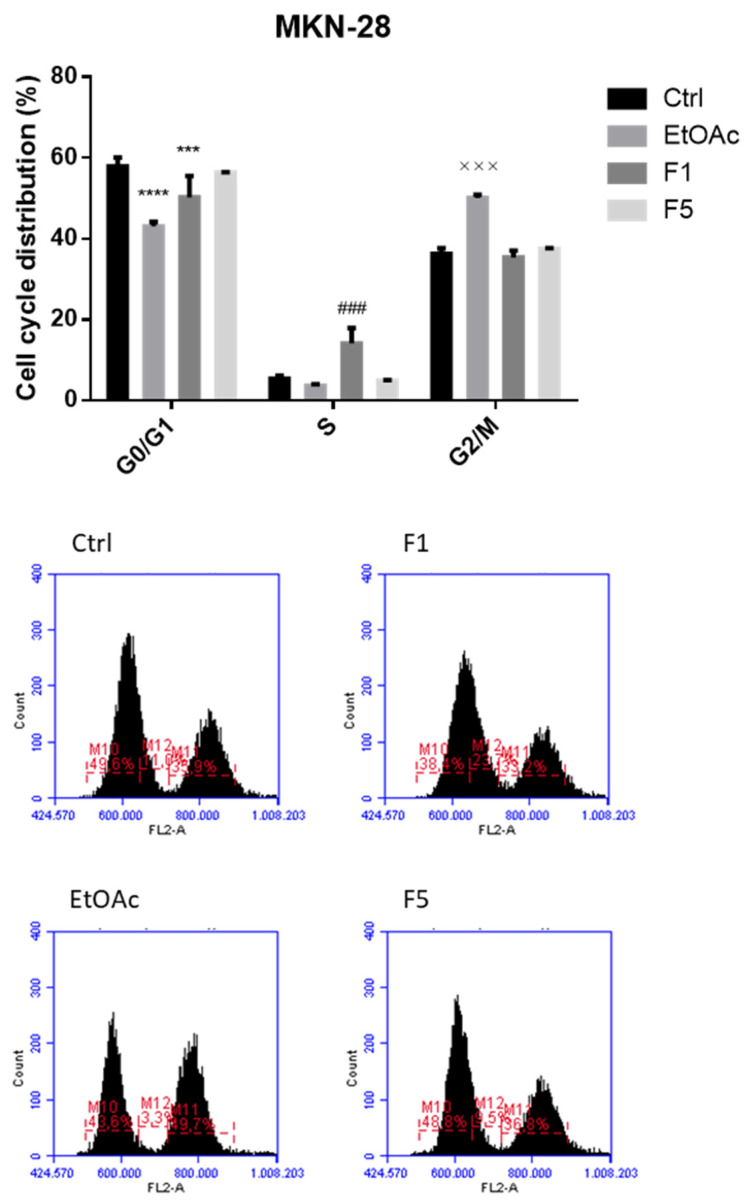
Effect of *F. vesiculosus* samples on the cell cycle distribution of MKN-28 cells after 48 h. Data are expressed as percentage of propidium iodide positive (PI^+^) cells and are presented as the mean ± SEM of at least three independent experiments (*** *p* < 0.001, **** *p* < 0.0001 when compared with control; the symbols *, #, and × were used to express statistical significance within G0/G1, S, and G2/M, respectively).

Defects in cell cycle checkpoints are associated with an uncontrolled cellular proliferation, thus justifying why targeting the cell cycle represents an important strategy for cancer therapy. In this context, considering the promising cytotoxic effects of EtOAc, F1, and F5 observed in MTT assay, these three samples were selected for further evaluation of the possible alterations in the cell cycle of either gastric (MKN-28) or colon (Caco-2) cancer cells. For that, cells were incubated with each sample at their IC_50_ concentrations as previously determined in the MTT assay, and subsequently analyzed for the distribution of G0/G1 (resting/growth phase), S (DNA synthesis phase), and G2/M (preparation for mitosis/mitosis phase). As depicted in Figure 2 and Figure 3, an arrest of the cell cycle at the S phase was observed in both cell lines when treated with F1, with this effect being more pronounced on MKN-28. On the contrary, EtOAc did not affect the cell cycle distribution of Caco-2 cells, although it revealed the capacity to interfere with that of MKN-28 by significantly decreasing the number of cells at the G0/G1 (43.0 ± 0.6%) phase and concomitantly increasing the cell counts at the G2/M phase (50.3 ± 0.4%) compared with the control (57.9 ± 1.1 and 36.2 ± 0.7%, respectively). Interestingly, F5 did not cause significant changes on the cycle of MKN-28 and Caco-2 cells. Based on these results, it is feasible to suggest that the capacity of EtOAc and F1 to inhibit cell proliferation occurs, at least partly, through cell cycle arrest, although the former only exhibited this capacity on MKN-28 cells. On the other hand, despite that F5 displayed the most promising cytotoxic effect in MTT assay, it does not seem to affect the cell cycle, suggesting that it might be promoting cytotoxicity via other mechanisms.

Previous studies have reported interesting results regarding the effect of brown algae molecules on the cell cycle of tumor cells. Indeed, cell cycle arrest at G0/G1 phase was described for numerous tumor cell lines treated with fucoidans extracted from different brown seaweed species [25], while fucoxanthin was shown to induce not only cell cycles arrest at the G0/G1 phase, but also at the G2/M phase in multiple tumor cell lines [26]. Although less explored, interesting results have also been reported for phlorotannins that have also been tested on several different tumor cell lines [27]. This is the case of dieckol isolated from *E. cava*, which was shown to induce the cell cycle arrest of both MCF-7 and SK-BR-3 breast cancer cells at the G2/M phase [28], while the addition of phloroglucinol to HT-29 cells over 24 h caused a dose-dependent increase of the cell counts at the G0/G1 phase [29]. Moreover, Geisen et al. [10] addressed the effect of a hydrophilic fraction isolated from a *F. vesiculosus* 70% acetone extract on two different pancreatic cancer cell lines (Colo357 and Panc89), demonstrating that, after 24 h of exposure, both cell lines evidenced a significant increase on the cell counts at the G2/M phase. No previous studies have, however, addressed the effect of *F. vesiculosus* phlorotannins on the cell cycle of gastric or colon cancer cell lines.

### 2.3. Apoptosis/Necrosis Detection on Cells Treated with F. vesiculosus Samples

**Figure 4:**
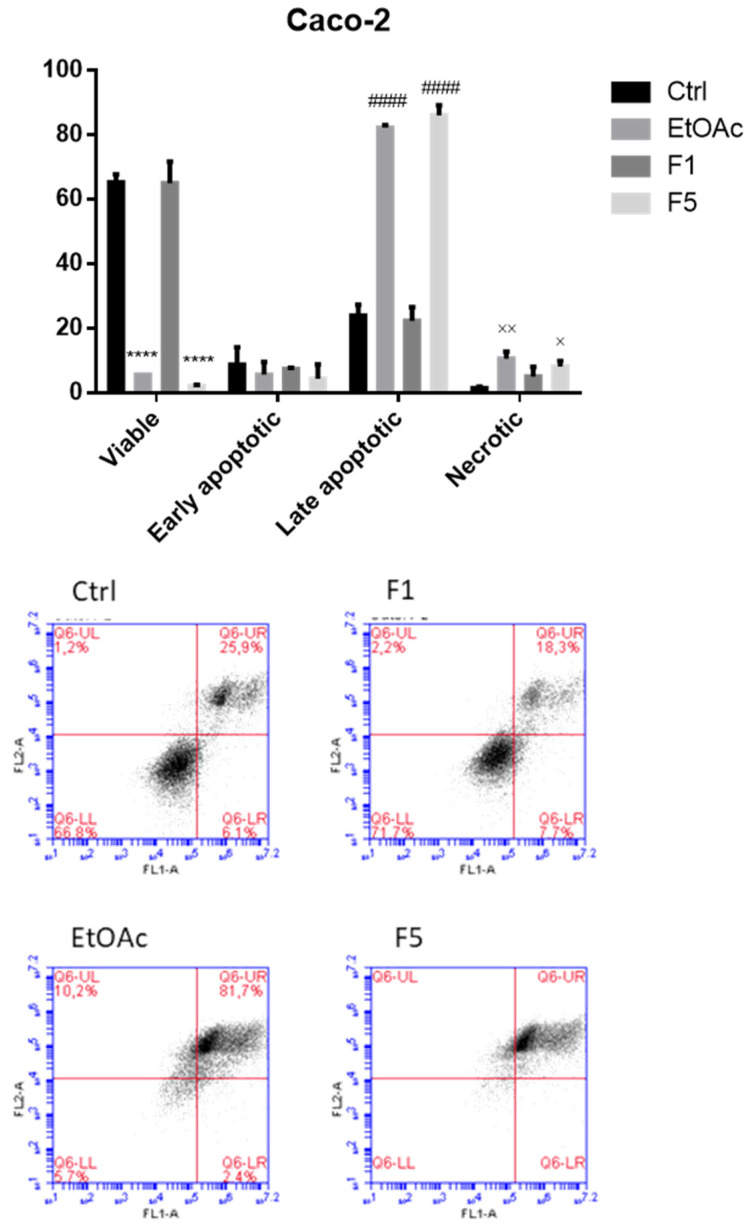
Detection of apoptosis/necrosis after 48 h treatment with *F. vesiculosus* samples via annexin V-FITC/PI labelling. The populations of early apoptotic cells, late apoptotic cells, necrotic cells, and viable cells were evaluated as a percentage of total cells and are presented as the means ± SEM of at least three independent experiments (* *p* < 0.05, ** *p* < 0.01, **** *p* < 0.0001 when compared with control; the symbols *, #, and × were used to express statistical significance within the viable, apoptotic, and necrotic cells, respectively).

**Figure 5:**
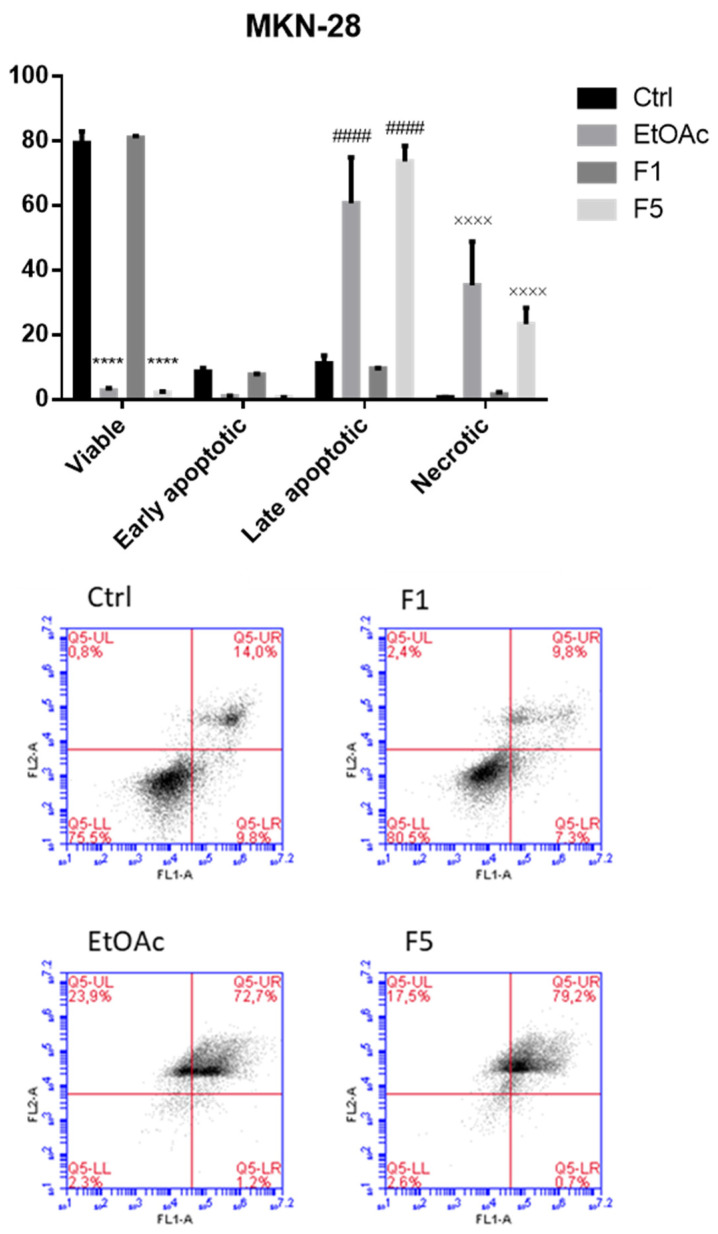
Detection of apoptosis/necrosis after 48 h treatment with *F. vesiculosus* samples via annexin V-FITC/PI labelling. The populations of early apoptotic cells, late apoptotic cells, necrotic cells, and viable cells were evaluated as a percentage of total cells and are presented as the means ± SEM of at least three independent experiments (**** *p* < 0.001 when compared with control; the symbols *, #, and × were used to express statistical significance within the viable, apoptotic, and necrotic cells, respectively).

To evaluate cell death and discriminate whether the exposure to *F. vesiculosus* samples triggers apoptosis and/or necrosis, cells were incubated under the same conditions used for the evaluation of the cell cycle and then stained with annexin V-FITC/PI. As illustrated in Figure 4 and Figure 5, after 48 h of exposure to EtOAc or F5, Caco-2 cells exhibited a significant increase in the percentage of the annexin V^+^/PI^+^, representing the late apoptotic cells (82.3 ± 0.4 and 86.1 ± 1.6%, respectively, compared with 23.9 ± 1.7% in the control), followed by a significant increase of annexin V^−^/PI^+^, representing the necrotic cells (10.7 ± 1.1 and 8.2 ± 0.9%, respectively, compared with 1.5 ± 0.2% in the control). Identical observations were recorded in MKN-28 cells, which also showed a great increase in the levels of late apoptotic cells exposed to EtOAc and F5 (60.8 ± 7.1 and 73.7 ± 2.4%, respectively, compared with 11.2 ± 1.3% in the control) and an even more pronounced increase of the percentage of necrotic cells (35.4 ± 6.8 and 23.3 ± 2.6%, respectively, compared with 0.8 ± 0.03% in the control).

These results are in line with previous works that highlighted the ability of phlorotannins and/or phlorotannin extracts from other seaweeds to induce necrosis and/or apoptosis on multiple tumor cell lines [9,30,31] and even in vivo [6]. For instance, dieckol isolated from *E. cava* was shown to trigger early and late apoptosis in SKOV3 ovarian cancer cells [9], MCF-7 breast cancer cells [28], and A549 non-small lung cancer cells [13], while induction of both early and late apoptosis was reported in Panc89 pancreatic cancer cells exposed to a hydrophilic fraction isolated from a *F. vesiculosus* 70% acetone extract for 24 h [10]. Induction of necrosis is, however, a mechanism that is not usually observed for these compounds, although two studies carried out with *Cystoseira* seaweeds reported a similar behavior. In more detail, the exposure of MCF-7 breast cancer cells to *C. barbata* 70% acetone extract over 48 h caused a dose-dependent increase not only in the number of apoptotic cells (both early and late), but also in the necrotic cells [31], while in HL60 and THP-1 cells, the treatment with *C. tamariscifolia* methanol extracts significantly increased the percentages of late apoptotic and necrotic cells, but not the percentages of the early apoptotic cells [32].

### 2.4. Characterization of Phlorotannins

The next step was to ascertain the phlorotannin profile of the most active *F. vesiculosus* samples and verify the presence of compounds that could be contributing to the effects described above.

According to our previous research works, the phlorotannin profile of EtOAc is of great complexity, revealing a wide spectrum of compounds that included fucols (e.g., tetrafucol, hexafucol), fuhalols (e.g., hydroxytetrafuhalol), and several fucophlorethol structures with different polymerization degrees, reaching at least 22 phloroglucinol units [33]. In turn, a phloroglucinol dimer with *m/z* at 247 was detected as a major compound present in F1 (data not shown). This compound, which was also detected on EtOAc, is a precursor of eckol, a phlorotannin compound that was previously documented as an inhibitor of the cell cycle of Reg3A-stimulated SW1990 pancreatic cancer cells at the G0/G1 phase [34].

**Figure 6:**
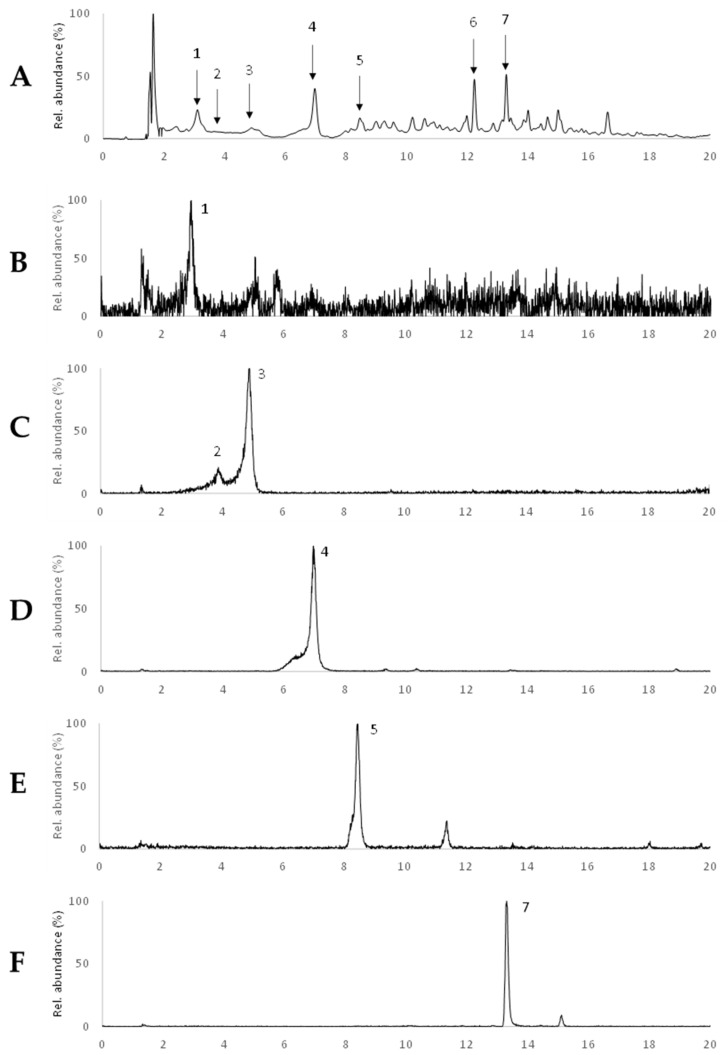
UV chromatograms recorded at 280 nm (**A**) and extracted ion chromatograms (EICs) of the major compounds detected in F5, namely *m/z* at 621 (**B**) *m/z* at 745 (**C**), *m/z* at 479 (**D**), *m/z* at 369 (**E**) and *m/z* at 507 (**F**). Peaks marked with numbers correspond to the tentatively identified compounds listed in Table 1.

A more detailed analysis was conducted on the subfraction F5 as this exhibited the best overall activity, i.e., evidenced the highest cytotoxic effect and the highest induction of apoptosis/necrosis in the different cell lines tested. Figure 6 shows the UV chromatogram recorded at 280 nm as well as the extracted ion chromatogram (EIC) of the major compounds detected. Overall, after the initial peak corresponding to the dead volume, seven different compounds were detected in this subfraction, presenting well-defined and abundant ions in their respective EICs, although in the chromatogram recorded at 280 nm, they did appear with low intensity. From these compounds, it was possible to identify one phloroglucinol trimer (peak 5), one tetramer (peak 4), one pentamer (peak 1), two hexamers (peaks 2 and 3), and two other phlorotannin derivatives (peaks 6 and 7).

**Figure 7:**
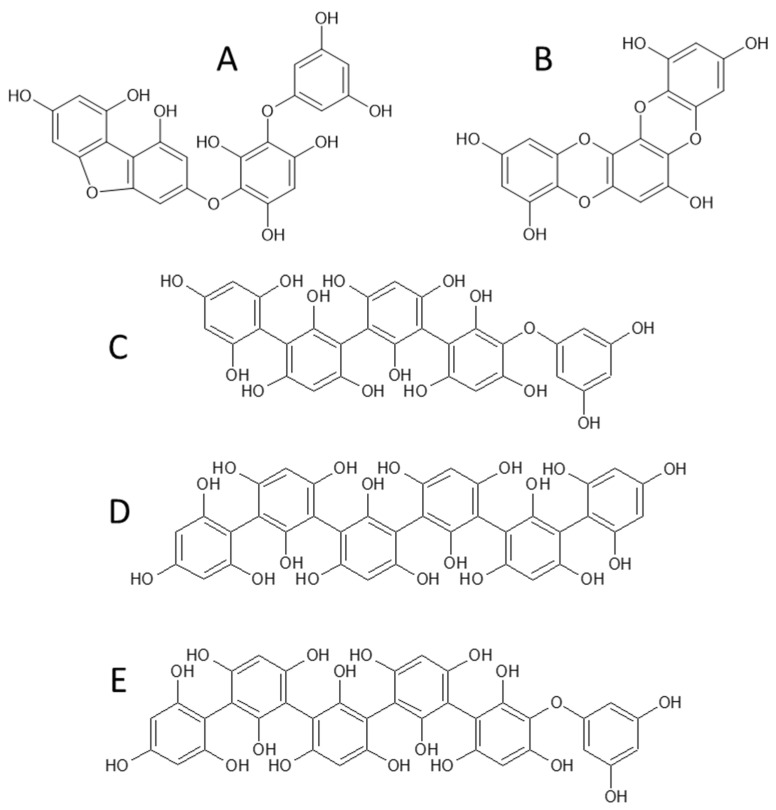
Chemical structure of the compounds tentatively identified in F5: (**A**) eckstolonol, (**B**) fucofurodiphlorethol, (**C**) trifucophlorethol, (**D**) hexafucol, and (**E**) tetrafucophlorethol.

In more detail, the proposed structure assigned to the trimer eluting at 8.5 min was eckstolonol (Figure 7A), according to its deprotonated molecular ion at *m/z* 369, followed by a fragmentation pattern typical of phlorotannins, with common losses of −18 Da, −44 Da, and −124 Da, and consistent with that previously reported in other works [35]. The appearance of this compound (also known as dioxinodehydroeckol) is more frequently associated with species from the genus *Ecklonia* [36,37,38], although it has been previously detected in Fucales, including in two species of the genus *Fucus,* namely *F. spiralis* and *F. guiryi* [35,39]. To the best of our knowledge, eckstolonol has not been previously reported in *F. vesiculosus,* although one eckstolonol derivative (MW 464 Da) was described in an aqueous extract of this macroalgae species [40].

The compound eluting at 7.0 min presented a deprotonated molecular ion at *m/z* 479, indicating a dehydroxylated phloroglucinol tetramer. This further fragmented into the product ions at *m/z* 461, corresponding to the loss of water, at *m/z* 435, which results from the internal cleavage of benzene ring structures and elimination of 44 Da; at *m/z* 353, as a consequence of the elimination of a phloroglucinol unit (PGU); and other fragments such as *m/z* 417 or *m/z* 313, which are resultant from the combination of the previous losses (−44−18 and −PGU−44, respectively). A compound presenting a similar retention time as well as an identical MS spectrum has been described previously as fucofurodiphlorethol (Figure 7B) owing to its structural resemblance to fucofuroeckol ([M − H]^−^ at *m/z* 477), except for the absence of the dioxin ring between the two inner phloroglucinol moieties, which would explain the 2 Da difference between their deprotonated ions. Although this compound is not usually described in the literature, recent works reported its presence in two different *F. vesiculosus* extracts, namely a microwave-assisted extract (using 57% ethanol) [41] and a conventional solid-liquid extract (using 70% acetone) [33].

The compounds eluted at 3.1 min ([M − H]^−^ at *m/z* 621) and 4.9 min ([M − H]^−^ at *m/z* 745) also presented retention times and MS spectra similar to those of trifucophlorethol (Figure 7C) and hexafucol (Figure 7D), assigned in our previous work [33], respectively. Interestingly, the isomer of the compound with a [M − H]^−^ at *m/z* 745 that eluted at 3.9 min revealed a fragmentation pattern slightly different from the one eluting at 4.9 min. In this case, the formation of the product ions such as *m/z* 619 and 601 denotes the loss of a phloroglucinol moiety and a phloroglucinol plus water, respectively, which is indicative of the presence of an ether linkage in the structure [35]. Therefore, this hexamer (eluting at 3.9 min) was tentatively assigned to a tetrafucophlorethol (Figure 7E).

Two additional compounds were detected at 12.2 and 13.3 min. The latter showed a [M − H]^−^ at *m/z* 507, presenting an MS/MS spectrum that was very similar to that described by Catarino et al. [42], evidencing several product ions such as *m/z* 277, 229, 245, or 261 (corresponding to the loss of fucol moiety, a fucol moiety itself, a phloroglucinol dimer, and loss of a phloroglucinol dimer, respectively) that strongly suggest that this is a phlorotannin derivative, even though its precise identification was not possible to achieve. Interestingly, although such a compound has not yet been described in the literature, recent works of our research group found a compound with the same deprotonated molecular ion and fragmentation pattern on a microwave-assisted ethanolic extract from *F. vesiculosus* [41]. Likewise, although the identification of the compound eluting at 12.2 min ([M − H]^−^ at *m/z* 611) was not achieved, its MS/MS spectrum presented some product ions including *m/z* 593 (−18), 567 (−44), and 469 (−*O*−phloroglucinol) that are typical of phlorotannin compounds, thus allowing to suggest that this might be a phlorotannin derivative as well.

Notably, among the compounds detected, eckstolonol has been described as a good antitumor agent against MCF-7 breast cancer cells, causing a dose-dependent anti-proliferative effect that was resultant from the induction of apoptosis via activation of the expression of p53, Bax, caspase-3, and caspase-9, alongside the decreased expression of Bcl-2 [12]. Moreover, phlorofucofuroeckol-A, a derivative of fucofuroeckol that has close structural similarities to fucofurodiphlorethol, was also reported to induce apoptosis in several human colorectal cancer cell lines (HCT116, SW480, LoVo, and HT-29 cells) via activation of the expression of ATF3, a protein that is tightly correlated to the induction of apoptosis in colorectal cancer [43]. Therefore, the presence of these two compounds in F5 could be one of the possible explanations for the remarkable increase of the levels of apoptotic cells observed in the annexin V-FITC/PI-stained cells (Section 2.3).

## 3. Materials and Methods

### 3.1. Chemicals

Grounded *F. vesiculosus* samples from July 2017 were purchased from Algaplus Lda. Acetone, ethanol, methanol, *n*-hexane, ethyl acetate, acetonitrile, dimethyl sulfoxide, and Nonidet P-40 were acquired from Fisher (Pittsburgh, PA, USA). Formic acid, PBS reagents (sodium salt, sodium chloride, potassium chloride, disodium hydrogen phosphate, and potassium dihydrogen phosphate), sodium citrate, antibiotic/antimycotic solution (100 units/mL of penicillin, 10 mg/mL of streptomycin, and 0.25 mg/mL of amphotericin B), trypsin, Roswell Park Memorial Institute (RPMI) 1640 medium, and DMEM were purchased from Sigma (St. Louis, MO, USA). Propidium iodide (PI), RNAse, and annexin V-FITC were acquired from Immunostep (Salamanca, Spain), while FBS was purchased from Lonza (Verviers, Belgium) and 3-(4,5-dimethylthiazol-2-yl)-2,5-diphenyltetrazolium bromide (MTT) from Himedia Laboratories (Einhausen, Germany). All reagents were of analytical grade or of the highest available purity.

### 3.2. Extraction and Purification of Phlorotannins from F. vesiculosus

Extraction and solvent partitioning were performed following the optimized procedure described previously [42]. Briefly, 100 g of dried algal powder was dispersed in 7000 mL of 70% acetone solution for 3 h at room temperature (RT) under constant agitation and filtered through a G4 glass filter, yielding a crude extract (CRD) with a total phlorotannin content of 10.7 ± 1.5 mg PGE/g extract. Afterwards, the extract was defatted with *n*-hexane (1:1 *v/v*, three times) and the aqueous layer was extracted with ethyl acetate (1:1 *v/v*, three times), yielding an ethyl acetate-soluble fraction (EtOAc) with a phlorotannin content of 17.1 ± 1.5 mg PGE/g extract. To obtain subfractions of different molecular weights, this EtOAc-soluble fraction was further submitted to gel filtration on a Sephadex LH-20 column according to the procedure reported by Wang et al. [17], using solvents of decreasing polarity eluting stepwise, namely, aqueous methanol 50% (*v/v*), aqueous methanol 75% (*v/v*), pure methanol, methanol and acetone 3:1 (*v/v*), methanol and acetone 1:1 (*v/v*), and finally methanol and acetone 1:3 (*v/v*). From this gel filtration, nine subfractions were recovered (F1—267.9 mg, F2—30.8 mg, F3—82.3 mg, F4—10.1 mg, F5—10.1 mg, F6—9.4 mg, F7—72.1 mg, F8—40.5 mg, and F9—9.0 mg), and the solvents were evaporated under reduced pressure prior to lyophilization and storage at −20 °C.

### 3.3. Cell Culture

Three human cancer cell lines, MKN-28 (human stomach adenocarcinoma, Cell Bank, Riken BioResource Center, Tsukuba, Japan), HT-29 (human colorectal adenocarcinoma, INSERMU178, Villejuif, France), and Caco-2 (human colorectal adenocarcinoma, ATCC, Manassas, VA, USA), were grown as monolayers from passage number 30 to 50 and maintained at 37 °C in an atmosphere of 5% CO_2_. As a non-cancer model, HFF-1 (human foreskin fibroblasts, ATCC, USA) were used. HFF-1 cells were cultured in 22.1 cm^2^ plates between passages number 4 and 9. For routine maintenance, the cells were cultured in 22.1 cm^2^ plates as monolayers and maintained in RPMI-1640 (MKN-28) or DMEM (HT-29, HFF-1, and Caco-2), supplemented with 10% (MKN-28, HFF-1) or 15% (Caco-2 and HT-29) fetal bovine serum (FBS) and 1% of antibiotic/antimycotic solution (100 units /mL of penicillin, 10 mg/mL of streptomycin and 0.25 mg/mL of amphotericin B). The medium was replaced every two days and the cells were harvested every two weeks.

### 3.4. Cell Viability

The effect of each sample on cell viability was evaluated according to the MTT assay as previously described [44]. For this assay, 100 μL of cell suspensions (1.5 × 10^5^ cells/mL) were plated in 96-well plates and allowed to stabilize overnight at 37 °C under a humidified atmosphere with 5% CO_2_. The effect of the vehicle solvent (DMSO) was evaluated in all experiments by exposing untreated control cells to the maximum concentration (0.1%) of DMSO used in each assay. A stock solution of the studied samples was prepared in DMSO and kept at −20 °C, and appropriate dilutions of each sample were freshly prepared just prior to every assay. Cells were then exposed to the respective samples, with a maximal solvent concentration of 0.1% DMSO, and incubated for 48 h. Afterwards, the medium was discarded, and the cells were washed with PBS prior to the addition of MTT solution at 0.45 mg/mL. The crystals of formazan were then allowed to form for 1.5 h and subsequently solubilized with DMSO prior to recording the absorbance at 570 nm on a standard spectrophotometer (Biotek PowerWave XS, BioTek Instruments Inc., Winooski, VT, USA). The results were expressed relative to untreated cells’ viability.

### 3.5. Flow Cytometric Analysis of the Cell Cycle and Apoptosis via Annexin V-PI Double Staining

Caco-2 and MKN-28 cells at exponential growth were obtained by plating 1.5 × 10^6^ cells/mL in a six-multiwell plate followed by 24 h incubation. Afterwards, the medium was replaced with fresh medium (control) or fresh medium supplemented with *F. vesiculosus* samples at the IC_50_ concentration previously determined through the MTT assay. After 48 h of incubation, cells were trypsinized, collected, and centrifuged for 5 min at 500 g, and the pellets were washed twice with PBS. For the cell cycle analysis, cells were fixed with ice cold 70% ethanol for 30 min followed by staining with PI buffer (0.05 mg/mL PI, 0.02 mg/mL of RNAse, 0.2% *m/v* of Nonidet P40, and 0.1% m/v sodium citrate in water) for 4 h at a temperature of 4 °C. Finally, the samples were analyzed on a BD Accuri C6 flow cytometer (BD Biosciences, Franklin Lakes, NJ, USA). In all experiments performed, at least 10 × 10^3^ events were acquired, and the experimental data were analyzed using the BD Cytometric Software (version 16 2.1).

For apoptosis analysis, cells were incubated with 5 μg/mL annexin V-FITC and 1 μg/mL PI for 15 min at room temperature in the dark, and then analyzed using the same apparatus.

### 3.6. UHPLC-DAD-ESI/MS Analysis

Chromatographic analysis was carried out as reported previously by Catarino et al. [42] for the fraction that showed the most promising anti-tumor activity in an Ultimate 3000 (Dionex Co., San Jose, CA, USA), an apparatus consisting of an autosampler/injector, a binary pump, a column compartment, and an ultimate 3000 Diode Array Detector (Dionex Co., San Jose, CA, USA), coupled to a Thermo LTQ XL (Thermo Scientific, San Jose, CA, USA) ion trap mass spectrometer equipped with an ESI source. The LC separation was carried out in a Hypersil Gold (ThermoScientific, San Jose, CA, USA) C18 column (100 mm length; 2.1 mm i.d.; 1.9 µm particle diameter, end-capped) maintained at 30 °C and a binary solvent system composed of (A) acetonitrile and (B) 0.1% of formic acid (*v/v*). The solvent gradient started with 5–40% of solvent (A) over 14.72 min, from 40–100% over 1.91 min, remaining at 100% for a further 2.19 min, before returning to the initial conditions. The flow rate was 0.2 mL/min and UV/vis spectral data for all peaks were accumulated in the range of 200–700 nm, while the chromatographic profiles were recorded at 280 nm. Control and data acquisition of MS were carried out with the Thermo Xcalibur Qual Browser data system (ThermoScientific, San Jose, CA, USA). Nitrogen above 99% purity was used, and the gas pressure was 520 kPa (75 psi). The instrument was operated in negative mode with the ESI needle voltage set at 5.00 kV and an ESI capillary temperature at 275 °C. The full scan covered the mass range from *m/z* 100 to 2000. CID–MS/MS experiments were performed for precursor ions using helium as the collision gas with a collision energy of 25–35 arbitrary units. All solvents used were of LC-MS grade.

### 3.7. Statistical Analysis

Data were expressed as mean ± SEM of three similar and independent experiments and analyzed using one-way analysis of variance (ANOVA) followed by Dunnet’s post hoc test. The statistical tests were applied using GraphPad Prism, version 7.00 (GraphPad Software, San Diego, CA, USA) and the significance level was *p* < 0.05.

## 4. Conclusions

**Figure 8:**
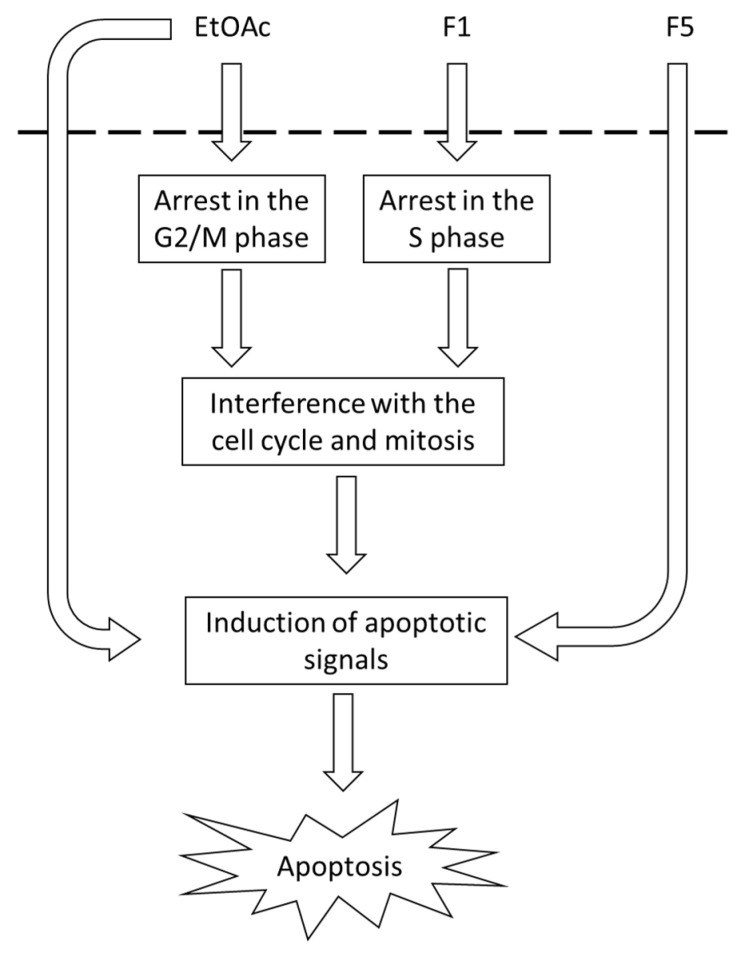
Schematic representation of the antitumor mechanism of *F. vesiculosus* phlorotannin-rich extract and subsequent fractions in the cell lines tested (Caco-2 and MKN-28).

In this work, *Fucus vesiculosus* phlorotannins were shown to hold potential as antitumor agents against both gastric and colon cancer because some of its fractions were capable of inducing selective cytotoxic effects towards tumor cell lines, but not on normal cell lines. Among the 11 samples tested, those presenting the most promising effects were EtOAc, F1, and F5. The collected data suggest that F1 antitumor activity might result in part from induction of cell cycle arrest at the S phase, while F5 exerts its effect via a remarkable stimulation of apoptosis and necrosis. Likewise, EtOAc antiproliferative effects on MKN-28 cells were resultant of both cell cycle arrest (on G2/M phase) and induction of apoptosis/necrosis, while on Caco-2 cells, it did not interfere with the cell cycle, but strongly triggered apoptosis and necrosis (Figure 8).

The UHPLC-DAD-ESI-MS^n^ analysis of the most active sample, i.e., F5, allowed to detect seven phlorotannin compounds, of which eckstolonol and fucofurodiphlorethol might be important contributors for the anti-proliferative effects displayed by this sample.

In summary, the results reported herein contribute to a better understanding of the mechanisms behind the antitumor properties of *F. vesiculosus* phlorotannins, although further work would be necessary to better comprehend the intracellular mechanisms that are being affected by the presence of these compounds. Therefore, the next step is to address the expression of anti- and pro-apoptotic proteins, as well as necrosis-related proteins and other relevant proteins involved in the pathophysiology of colon and gastric cancer.

## Figures and Tables

**Table 1:** Tentative assignment of the compounds detected in the F5 analyzed by UHPLC-ESI-MS/MS.

Peak	RT (Min)	[M − H]^−^ (*m/z*)	MS/MS Ions (-Loss) *	Tentative Assignment
1	3.1	621	**603** (−18), 585 (−36), 455 (−166), 331 (−290), 559 (−44–18), 577 (−44), 519 (−102), 289 (−332), 429 (−192), 495 (−PGU), 477 (−PGU–18), 411 (−PGU–84)	Trifucophlorethol
2	3.9	745	**727** (−18), 709 (−36), 455 (−PGU−166), 579 (−166), 289 (−3PGU−84), 701 (−44), 683 (−44−18), 643 (−84−18), 661 (−84), 331, 437 (−PGU−166−18), 601 (−PGU–18), 619 (−PGU)	Tetrafucophlorethol
3	4.9	745	**727** (−18), 455 (−PGU−166), 579 (−166), 709 (−36), 701 (−44), 289 (−3PGU−84), 683 (−44−18), 643 (−84−18), 437 (−PGU−166−18), 411 (−334), 429 (−316), 553 (−192), 433 (−2PGU−44−18)	Hexafucol
4	7.0	479	**461** (−18), 435 (−44), 417 (−44–18), 391 (−88), 313 (−166), 349 (−130), 353 (−126)	Fucofurodiphlorethol
5	8.5	369	**325** (−44), 341 (−28), 299 (−70), 351 (−18), 297 (−72), 281 (−88), 245 (−124)	Eckstolonol
6	12.2	611	**565** (−46), 593 (−18), 567 (−44), 579 (−32), 469 (−142), 356 (−255), 551 (−60), 243 (−368)	Phlorotannin derivative
7	13.3	507	**489** (−18), 277 (−230), 229 (−PGU−108−44), 461 (−46), 463 (−44), 479 (−28), 445 (−44–18), 275 (−232), 261 (−246), 245 (−262), 421 (−86), 297 (−PGU−84)	Phlorotannin derivative

* Fragments are arranged in descending order of relative abundance, with bold values highlighting the most abundant fragment. RT—retention time, PGU—phloroglucinol unit.

## Data Availability

Data is available from the corresponding author.
